# The First Record of *Argulus foliacesus* (Crustacea: Branchiura) Infestation on Lionhead Goldfish (*Carassius auratus*) in Iran

**Published:** 2010-06

**Authors:** V Noaman, Y Chelongar, AH Shahmoradi

**Affiliations:** Department of Veterinary, Esfahan Research Center for Agriculture and Natural Resources, Iran

**Keywords:** *Argulus foliaceus*, lionhead goldfish, Ectoparasite, Iran

## Abstract

*Argulus foliaceus* (Crustacea: Branchiura), or the fish louse, is an ectoparasite of the skin or gill of the fresh water fish species. Clinical signs in infected fish include scratching on aquarium walls, erratic swimming, and poor growth. It causes pathological changes due to direct tissue damage and secondary infections. In the present study, lionhead goldfish *(Carassius auratus*), taken from a goldfish aquarium with symptoms such as abnormal swimming, poor growth and death, were examined for ectoparasites. The parasites collected from the skin and fins of fish were identified as *A. foliaceus*. Then, treatment was carried out by trichlorfon. After administration, no parasite was observed on the fish. This is the first report of infection with *A. foliaceus* of lionhead goldfish (*Carassius auratus)* in Iran.

## Introduction

The most common members of the Branchiura belong to the genus *Argulus,* so called fish lice. Many of the species are parasitic on marine fishes, and about 15 spp. are found on freshwater fishes (1, 2).

It is sometimes possible to see the parasites with a naked eye because these parasites are 5–10 mm in size and consist of a head, thorax, and abdomen. The head is covered by a flattened horseshoe-shaped carapace, maxillipeds, peroral sting, and basal glands. The thorax has four segments, each bearing a pair of swimming legs. The abdomen is a simple bilobed segment. When seen from the dorsal aspect, two prominent movable compound eyes are visible in the head region (2, 3).

This parasite has a direct life cycle (4). Mating takes place during the free-swimming stage and mature females leave the host and lay several hundred eggs on vegetation and various objects in the water. Eggs are ovoid in shape and are covered by a gelatinous capsule. Depending on the temperature, 40–100 days are required for completion of the life cycle. After being hatched the parasite must find a suitable host within four days, otherwise it will die. Adults may live free from the host for up to 15 days (2).The mouthparts of *Argulus* are greatly reduced, and the most striking feature is the modification of the second maxillae into two suction cups by which the parasite holds onto its host (5).

When the parasite has attached itself to a fish, it will insert its needle-shaped mouth into the tissue. This parasite causes patches of swollen and bleeding skin and can affect the entire body, including fins and gills. It feeds on blood and other bodily fluids, and causes further harm to the fish by injecting digestive enzymes that can lead to systemic illness. Other symptoms are small dark spots on the skin, typically behind the fins and around the head. Besides the damage and stress caused by *Argulus* itself, one of the main worries for fish producers is the associated secondary infestation that can result from infestations with parasite. Several studies have examined the role of parasites as vectors for other diseases such as Aeromoniasis or Pseudomoniasis (1, 6).

The aim of this study was determination of causes of death in lionhead goldfish and treatment of infested fish as well.

## Case Report

In October 2008, a goldfish producer referred to Veterinary Department of Esfahan Research Center for Agriculture and Natural Resources, Esfahan, Iran. He complained of poor growth, abnormal swimming, and death in goldfish aquarium. Direct examination of fish showed small red spots on the skin, typically behind the fins and around the head thereafter body surface, head, gill, and fins were examined for ectoparasites.

**Fig. 1:**
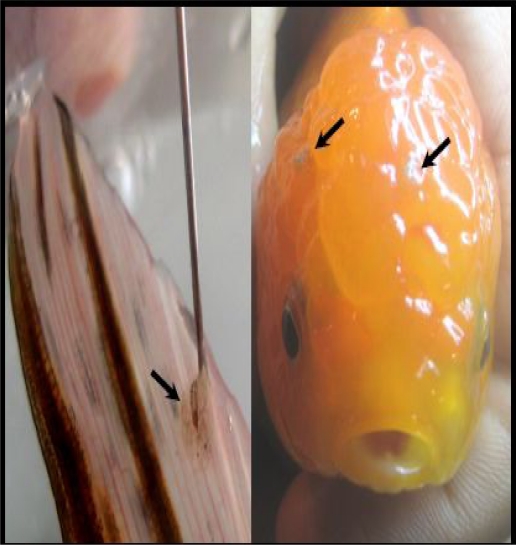
*Argulus foliaceus* on the tail fin and head of goldfish

**Fig. 2:**
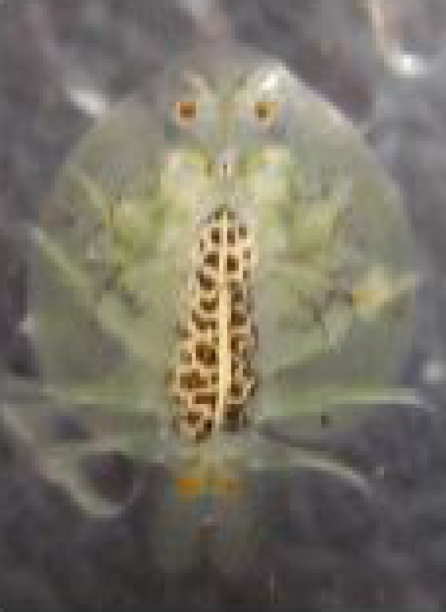
*Argulus foliaceus* with rounded abdominal lobes

**Fig. 3:**
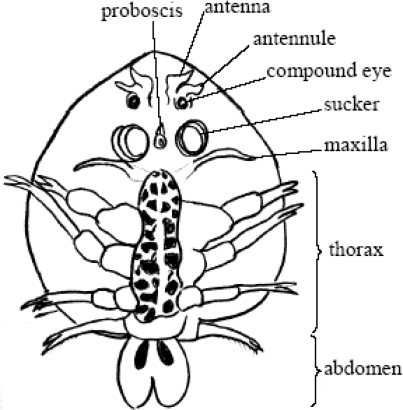
Schematic representation of *Argulus foliacesus* (Ventral view)

Out of 80 lionhead goldfish (*Carassius auratus*), 60 fish were found infested by *Argulus sp*. (Fig. 1). The prevalence of ectoparasite infestation in this fish appeared 75% and the mean number of parasites per fish was 2–3. The parasites were removed, fixed in 70% ethanol, and identified morphologically using the characteristics clues (2). The parasites were 4520–6560×2340–3560 µm in size. Under the light microscope, these parasites were identified at *Argulus foliaceus* according to the rounded lobes of abdomen and the posterior emargination not reaching the mid-line and posterior lobes cephalothoracic carapace not extended beyond the beginning of abdomen (Fig. 2, 3).

The owner was recommended to disinfect the aquariums and equipment to remove completely eggs, and treatment of fish with trichlorfon (0.25 mg/l at temperatures below 27 °C, or with 0.50 mg/l above 27 °C) (7). The bath was repeated twice a week and was found effective. During treatment, neither adverse effects nor mortality was observed throughout the trichlorfon bath. All fish were checked in terms of parasite following the treatment. No parasite was observed on the fish. Because the larvae emerged from eggs did not be affect from drug transformed into juvenile and then into adult stages, the symptoms were reappeared 3 weeks later and the drug administrated again at same dose. Although the source of contamination of the aquarium with *A. foliaceus* was not defined, marine sand contain live eggs was suspected.

## Discussion

*Argulus* sp. is reported from different fish species worldwide (7–12) and in Iran by Peygan 1999 (13). In the present study, for the first time *A. foliaceus* was reported from lionhead goldfish (*Carassius auratus)* in Iran.

Differentiation of *A. foliaceus* from other species such as *A. japonicus* and *A. coregoni* is necessary. *A. coregoni* has acuminate abdomen lobes and 12 mm body length, in *A. japonicus* posterior lobes of cephalothoracic carapace extended beyond the beginning of abdomen but *A. foliaceus* has rounded lobes of abdomen, the posterior emargination not reaching the mid-line and posterior lobes cephalothoracic carapace not extended beyond the beginning of abdomen (7).

*A. foliaceus* infestations cause the skin irritation manifested by flicking of the fins (1, 6). This is often accompanied by increased mucus production over the skin surface and the appearance of small haemorrhages (6). Pathogenic effects include skin damage to their hosts and manifest themselves as skin lesions (dermatitis) (14). These lesions could become secondarily infected by bacteria (7). The dermatitis is due to the damaging effect of the suckers and proboscis. Anemia is another significant pathological effect caused by feeding. In this study, abnormal swimming, rubbing themselves against the wall of aquarium and lack of appetite were observed in infested fish. The skin and fins have numerous reddish points and hemorrhagic areas. Hindle reported fish infested with *Argulus* were sluggish and isolated themselves in the corners of aquariums (15). In addition to the pathogenic effects mentioned above, *Argulus* is a vector of certain viruses, such as *Rhabdovirus carpio* or spring viraemia (16) and carp pox or viral epithelioma (17).

It is known that *Argulus* infestations lead to secondary parasitic infestation of the skin (1, 3). Some authors reported that *Costia necatrix* accompanied by *A. foliaceus* in infected fish, and *Trichodina* sp*., Trichodinella* sp. and *Apiosoma* sp. were observed in skin and gills preparation (1, 18). In this study, no other parasites were observed on the body surface and gill.

The number of parasite on each fish may be different. Fryer reported thousands of *Argulus* species occurring on a single tench (19). In this study, 2–3 *A. foliaceus* were counted on an individual lionhead goldfish. This might be related to the early stage of infection. Pathogenesis was not severe because these fish were big and a few parasites being found on the fish. Although *A. foliaceus* can easily be diagnosed on the skin of fish by direct examination, a heavy *A. foliaceus* infestation in small and young fish may cause death and the disease must be differentiated from the many other causes of death such as parasitic, viral, and bacterial diseases.

The treatments of *Argulus* infestations include the use of common chemicals such as salt (NaCl) (20). Other common chemicals used in experimentation include formaldehyde (21), potassium permanganate (2–5 mg/l bath) (22), and formalin (23). The most effective treatment against argulusosis is organophosphates (1). Organophosphates, usually 2–3 doses at one-week intervals, are needed to treat the emerging larvae and juveniles. Treatments such as trichlorfon (0.25 ppm for several hours) (24), and emamectin benzoate (25) have been used to eradicate *Argulus*. In all situations, we have to treat the entire aquarium equipment to get rid of *Argulus*. It is possible to pick off the parasite from the fish with a pair of forceps, but it can be tricky to find all the parasites and remove them.

The keys to prevention are avoidance and quarantine. Fish must closely be inspected for the presence of the parasite at the time of purchasing. In addition, eggs are laid on vegetation and other substrates and can be introduced into a pond or aquarium by plants, rocks, or other materials.
